# Identification of genetic variants for clinical management of familial colorectal tumors

**DOI:** 10.1186/s12881-018-0533-9

**Published:** 2018-02-20

**Authors:** Mev Dominguez-Valentin, Sigve Nakken, Hélène Tubeuf, Daniel Vodak, Per Olaf Ekstrøm, Anke M. Nissen, Monika Morak, Elke Holinski-Feder, Alexandra Martins, Pål Møller, Eivind Hovig

**Affiliations:** 1 Department of Tumor Biology, Institute for Cancer Research, Oslo University Hospital, Oslo, Norway; 2 Inserm-U1245, UNIROUEN, Normandie Univ, Normandy Centre for Genomic and Personalized Medicine, Rouen, France; 3 Interactive Biosoftware, Rouen, France; 4 Medizinische Klinik und Poliklinik IV, Campus Innenstadt, Klinikum der Universität München, Ziemssenstr. 1, Munich, Germany; 5 MGZ—Medizinisch Genetisches Zentrum, Munich, Germany; 6 Department of Human Medicine, Universität Witten, Herdecke, Germany; 7 Department of Medical Genetics, Oslo University Hospital, Oslo, Norway; 8 Department of Informatics, University of Oslo, Oslo, Norway; 9 Institute of Cancer Genetics and Informatics, Oslo University Hospital, Oslo, Norway

**Keywords:** Lynch syndrome, Gene panel testing, *CHEK2*, RNA splicing mutations

## Abstract

**Background:**

The genetic mechanisms for families who meet the clinical criteria for Lynch syndrome (LS) but do not carry pathogenic variants in the mismatch repair (MMR) genes are still undetermined. We aimed to study the potential contribution of genes other than MMR genes to the biological and clinical characteristics of Norwegian families fulfilling Amsterdam (AMS) criteria or revised Bethesda guidelines.

**Methods:**

The Hereditary Cancer Biobank of the Norwegian Radium Hospital was interrogated to identify individuals with a high risk of developing colorectal cancer (CRC) for whom no pathogenic variants in MMR genes had been found in routine diagnostic DNA sequencing. Forty-four cancer susceptibility genes were selected and analyzed by using our in-house designed TruSeq amplicon-based assay for targeted sequencing. RNA splicing- and protein-dedicated in silico analyses were performed for all variants of unknown significance (VUS). Variants predicted as likely to affect splicing were experimentally analyzed by resorting to minigene assays.

**Results:**

We identified a patient who met the revised Bethesda guidelines and carried a likely pathogenic variant in *CHEK2* (c.470 T > C, p.I157T). In addition, 25 unique VUS were identified in 18 individuals, of which 2 exonic variants (*MAP3K1* c.764A > G and *NOTCH3* c.5854G >A) were analyzed in the minigene splicing assay and found not to have an effect on RNA splicing.

**Conclusions:**

Among high-risk CRC patients that fulfill the AMS criteria or revised Bethesda guidelines, targeted gene sequencing identified likely pathogenic variant and VUS in other genes than the MMR genes (*CHEK2, NOTCH3* and *MAP3K1*). Our study suggests that the analysis of genes currently excluded from routine molecular diagnostic screens may confer cancer susceptibility.

**Electronic supplementary material:**

The online version of this article (10.1186/s12881-018-0533-9) contains supplementary material, which is available to authorized users.

## Background

Heredity represents a major cause of colorectal cancer (CRC) with at least 20% of the cases estimated to develop due to genetic factors and about 5% being linked to inherited variants in cancer-predisposing genes [1–4]. Currently, patients with CRC are referred to germline mismatch repair (MMR) testing based on the identification of high-risk phenotypic features (i.e. early age of onset, family history, clinical criteria), but beyond microsatellite instability (MSI) and MMR immunohistochemistry (IHC) testing for Lynch syndrome (LS), no systematic approach to hereditary risk assessment exists [5].

LS is caused by a defective MMR system due to presence of germline defects in at least one of the MMR genes, *MLH1, MSH2, MSH6, PMS2* or to deletions of the 3′ portion of the *EPCAM* gene [6]. LS is clinically classified according to the Amsterdam (AMS) criteria and/or the Bethesda guidelines, both relying in clinical information and family history. The Bethesda guidelines also take into account the MSI signature characteristic of MMR-deficient tumors [7–10]. LS patients have an increased lifetime risk of CRC (70–80%), endometrial cancer (50–60%), stomach cancer (13–19%), ovarian cancer (9–14%), cancers of the small intestine, the biliary tract and brain as well as carcinoma of the ureters and renal pelvis [11].

However, a high proportion of cases who meet the clinical criteria for LS (~ 60%) do not carry pathogenic variants in the MMR genes and have been reported as familial colorectal cancer type X (FCCTX) or Lynch-like syndrome (LLS) according to their MSI status [12–16]. The genetic mechanisms are undetermined in the majority of these families [14].

DNA sequencing (DNA-seq) studies using multigene panels have reported that as much as ~ 18% of patients diagnosed with CRC below the age of 50 years have pathogenic variants in several genes that are not traditionally associated with CRC (*ATM*, *CHEK2*, *BRCA1*, *BRCA2*, *CDKN2A* and *PALB2*) [5, 17]. Notably, there is a need to determine whether these variants contribute to hereditary CRC risk via the combination of low- and moderate-penetrance susceptibility alleles [5, 17, 18].

Given the high frequency and wide spectrum of pathogenic variants, it has been suggested that genetic counseling and testing with a multigene panel should be considered for all patients with early-onset CRC [17, 19–23]. Importantly, the identification of high-risk CRC patients is a major issue, because morbidity and mortality from CRC and extracolonic cancers in these patients and their relatives can be decreased by early screening and intensive surveillance [19, 24–26].

In an effort to discover inherited genetic variants that influence biological and clinical characteristics of familial CRC developed in unrelated high-risk patients, who previously tested negative for pathogenic variants in MMR genes, we examined 44 cancer associated genes using next generation sequencing (NGS), and applied minigene-based assay to analyze the impact of a subset of genetic variants on RNA splicing.

## Methods

### Study population

The Hereditary Cancer Biobank of the Norwegian Radium Hospital was used to identify unrelated high-risk CRC individuals from families that fulfilled the AMS criteria or the revised Bethesda guidelines [7–10, 27]. By the standard diagnostic clinical techniques, all study subjects were demonstrated not to carry pathogenic variants or large genomic rearrangements in MMR genes (*MLH1, MSH2, MSH6* or *PMS2*).

Ethical approval for the study was granted by the Norwegian Data Inspectorate and Ethical Review Board (ref 2015/2382). All examined patients signed an informed consent for their participation in the study.

### Targeted sequencing

Genomic DNA was isolated from peripheral blood samples and targeted sequencing was carried out using a TrueSeq amplicon based assay v.1.5 on a MiSeq apparatus, as previously described [28, 29]. The 44-gene panel used in this study includes genes associated with cancer predisposition as described in a prior study [28, 29].

### Sequencing data analysis

Paired-end sequence reads were aligned to the human reference genome (build GRCh37) using the BWA-mem algorithm (v.0.7.8-r55) [30]. The initial sequence alignments were converted to BAM format and subsequently sorted and indexed with SAMtools (v.1.1) [30]. Genotyping of single nucleotide variants (SNV) and short indels was performed by GATK’s HaplotypeCaller. Filtering of raw genotype calls and assessment of callable regions/loci were done according to GATK’s best practice procedures, as described more detailed previously [28].

Variants were annotated using ANNOVAR (version November 2015) [31] and were queried against a range of variant databases and protein resources, namely dbSNP (build 147) [32], 1000 Genome Project phase3 [33], Exome Aggregation Consortium (ExAC) (http://exac.broadinstitute.org, accessed August 2015) [34], Genome Aggregation Database (gnomAD) (http://gnomad.broadinstitute.org, accessed October 2017) [34], Norwegian Germline Variations Database (http://norgene.no/vcf-miner/, accessed October 2017), ClinVar (May 2016) [35], UniProt Knowledgebase (release March 2016) [36] and the Pfam protein domain database (v29, December 2015) [28, 37].

### Nomenclature and classification of genetic variants

The nomenclature guidelines of the Human Genome Variation Society (HGVS) were used to describe the detected genetic variants [38]. The recurrence of the identified variants was established by interrogating four databases (in their latest releases as of November 2016): the Leiden Open Variation Database (LOVD), the Universal Mutation Database (UMD), ClinVar and the Human Gene Mutation Database (HGMD). The variants were classified according to the 5-tier classification system into the following categories: class 5 (pathogenic), class 4 (likely pathogenic), class 3 (uncertain variants or variants of unknown significance, VUS), class 2 (likely not pathogenic) and class 1 (not pathogenic) [3].

### In silico analyses of VUS

Two types of bioinformatics methods were used to predict the impact of selected variants on RNA splicing. First, we used MaxEntScan (MES) and SSF-like (SSFL) to predict variant-induced alterations in 3′ and 5′ splice site strength, as described by Houdayer et al. 2012 [39], except that here both algorithms were interrogated by using the integrated software tool Alamut Batch version 1.5, (Interactive Biosoftware, http://www.interactive-biosoftware.com). For prediction of variant-induced impact on exonic splicing regulatory elements (ESR), we resorted to ΔtESRseq- [40], ΔHZei- [41], and SPANR-based [42] as described by Soukarieh et al. [43]. Score differences (Δ) between variant and wild-type (WT) cases were taken as proxies for assessing the probability of a splicing defect. More precisely, we considered that a variant mapping at a splice site was susceptible of negatively impacting exon inclusion if ΔMES≥15% and ΔSSFL≥5% [39], whereas an exonic variant located outside the splice sites was considered as a probable inducer of exon skipping if negative Δ scores (below the thresholds described below) were provided by all the 3 ESR-dedicated in silico tools. We chose the following thresholds: <− 0.5 for ΔtESRseq-, <− 10 for ΔHZei-, and < − 0.5 for SPANR-based scores. In addition, we evaluated the possibility of variant-induced de novo splice sites by taking into consideration local changes in MES and SSFL scores. In this case, we considered that variants located outside the splice sites were susceptible of creating a competing splice site if local MES scores were equal to or greater than those of the corresponding reference splice site for the same exon.

In silico protein impact predictions of missense variants were performed with Align-GVGD (the VUS were predicted as deleterious when the values were from C35 or higher), SIFT, and MAPP using Alamut Batch version 1.4.4 (Interactive Biosoftware) and additionally with PolyPhen-2 and MutationTaster [44–48].

### Cell-based minigene splicing assays

In order to determine the impact of selected exonic variants on splicing, we performed functional assays based on the comparative analysis of the splicing pattern of WT and mutant reporter minigenes, as follows. First, genomic regions containing the exon of interest (internal exons only) and at least 150 nucleotides of the flanking introns were amplified by PCR [49] using patients’ DNA as template and primers indicated in Additional file 1: Table S1. Next, representative minigenes were created by inserting the PCR-amplified fragments into a previously linearized pCAS2 vector [43]. All constructs were sequenced to ensure that no unwanted mutations had been introduced into the inserted fragments during PCR or cloning. Then, WT and mutant minigenes were transfected into HeLa cells grown in 12-well plates (at ~ 70% confluence) using the FuGENE 6 transfection reagent (Roche Applied Science). Twenty-four hours later, total RNA was extracted using the NucleoSpin RNA II kit (Macherey Nagel) and, the minigenes’ transcripts were analyzed by semi-quantitative RT-PCR using the OneStep RT-PCR kit (Qiagen), as previously described [43]. The sequences of the RT-PCR primers are shown in Additional file 1: Table S1. Later, RT-PCR products were separated by electrophoresis on 2.5% agarose gel containing EtBr and visualized by exposure to UV light under saturating conditions using the Gel Doc XR image acquisition system (Bio-Rad), followed by gel-purification and Sanger sequencing for proper identification of the minigenes’ transcripts. Finally, splicing events were quantitated by performing equivalent fluorescent RT-PCR reactions followed by capillary electrophoresis on an automated sequencer (Applied Biosystems), and computational analysis by using the GeneMapper v5.0 software (Applied Biosystems).

## Results

### Clinical characteristics and family history

Upon querying the Hereditary Cancer Biobank of the Norwegian Radium Hospital for cases that fulfill the AMS and/or the revised Bethesda guidelines, we identified 34 unrelated potential high-risk CRC individuals who did not carry pathogenic variants in MMR genes. The median age at first CRC diagnosis was 51.5 years (range: 34–86 years).

Pedigree information showed that 13 (38%) families fulfilled the AMS I and/or II criteria and the revised Bethesda guidelines while 21 (62%) met the revised Bethesda guidelines only (Table 1). Fifteen (44%) patients had tumors with MSI and/or MMR IHC data available, of which 2 (13%) were MSI-high and/or MMR deficient. Clinical, family and tumor data information is detailed in Table 1.

**Table 1:** Summary of International Classification of Diseases, 9th Revision (ICD9), gender, age at diagnosis, clinical criteria and tumor molecular characteristics of the familial CRC families

Patient_ID	Gender	ICD9 diagnosis (age)	AMS criteria	Revised Bethesda	Tumor molecular characteristics
3222	F	CC (54), Hyperplastic polyp (55/61/62/63/65), BC (70)	0	Y	MMR IHC proficient
3308	F	CC (43), BC (51/52)	0	Y	MMR IHC proficient
3387	F	BC (40), OC (70), CC (80)	0	Y	MMR IHC proficient
3426	M	MM (39)	I & II	Y	na
4932	F	CC (34), EC (40), Hyperplastic polyp (43), BT (46)	I & II	Y	na
5324	F	M (52), CC (59), SMC (na), BC (72)	0	Y	na
6174	F	Hyperplastic polyp (63/67), BC (65)	I & II	Y	MMR IHC proficient
6977	F	TC (66)	0	Y	MMR IHC proficient
9876	F	M (45), BC (54)	0	Y	na
9998	F	Hyperplastic polyp (45), CC (45)	II	Y	MMR IHC proficient
10,675	F	BC (51), Hyperplastic polyp (59), TC (60)	II	Y	na
12,954	F	Hyperplastic polyp (69), ML (70)	II	Y	na
13,072	M	Hyperplastic polyp (63/64/65), CC (65/67)	0	Y	na
14,930	F	Hyperplastic polyp (86), CC (86)	0	Y	MMR IHC proficient
18,843	F	BC (44), CC (49), SMC (na)	0	Y	na
19,411	M	PC (70)	0	Y	MSH6 IHC deficient
19,673	F	BC (40/42)	II	Y	na
20,612	F	Hyperplastic polyp (59/65), EC (70)	0	Y	na
21,368	F	OC (62)	0	Y	na
22,295	F	Hyperplastic polyp (53), M (58)	0	Y	na
23,761	F	Hyperplastic polyp (40/42/44), BC (50)	0	Y	na
23,910	F	M (43), Hyperplastic polyp (49), BC (63), BT (63)	0	Y	na
24,140	F	CC (45/67), BC (56)	0	Y	na
24,447	F	BC (57/66), CC (66)	0	Y	MLH1/PMS2 IHC deficient and MSI
11,705	F	THC (53), KC (53/63)	II	Y	MMR IHC proficient
12,673	F	OC (23), SMC (36), RC (62)	II	Y	na
13,393	M	RC (48), CST (58)	I & II	Y	MMR IHC proficient and MSS
14,963	F	Hyperplastic polyp (69), BC (62)	0	Y	na
19609^a^	F	CC (42), M (44), BC (57)	0	Y	MMR IHC proficient
22,953	F	BC (53)	II	Y	na
24,789	F	CC (43), RC (65), BC (72)	I & II	Y	MMR IHC proficient and MSS
25,167	M	CC (55)	I & II	Y	MMR IHC proficient
5597	M	Hyperplastic polyp (53/54), SC (55), KC (62)	0	Y	MMR IHC proficient
8913	F	Hyperplastic polyp (59), BC (61), TC (69)	0	Y	na

*CRC* colorectal, *ICD9 diagnosis* International Classification of Diseases, 9th Revision, *CC* colon cancer, *BC* breast cancer, *AMS* Amsterdam criteria, *0* not fulfill the AMS criteria, *Y* yes, *MMR* mismatch repair, *IHC* immunohistochemistry, *MSI* microsatellite instabily, *MSS* microsatellite stable, *na* not available, *OC* ovary cancer, *MM* multiple myeloma, *EC* endometrial cancer, *BT* brain tumor, *M* melanoma, *SMC* other malignant of the skin, *TC* trachea, bronchus, lung cancer, *ML* malignant neoplasms of lymphoid, *PC* prostate cancer, *THC* thyroid cancer, *KC* kidney cancer, *RC* rectum cancer, *CST* malignant neoplasm of connective and soft tissue, *SC* stomach cancer

^a^ Patient carrying *CHEK2* c.470 T > C, p.I157T

### Germline findings

Given that the families that fulfilled the AMS criteria and/or the Bethesda guidelines did not carry pathogenic variants in the MMR genes, we hypothesized that other genes could be implicated in the genetic determinism of these phenotypes.

In order to pursue this hypothesis, we collected DNA samples from all probands and performed high-throughput sequencing of a panel of 44 cancer-associated genes. For the 34 samples, mean depth of coverage ranged from 127 to 507 with the fraction of target bases with coverage ≥25 ranging from 80% to 93. The NGS results revealed that each individual carried an average of 26 SNV (between 19 and 33 per individual) in the set of 44 cancer susceptibility genes, most of which were common polymorphisms (allele frequency ≥ 1% in the general population) according to the ExAC database, and some being classified as benign or likely benign (class 1 or class 2) according to either ClinVar or the American College of Medical Genetics and Genomics (ACMG) guidelines [35, 50] (Table 2).

**Table 2:** Characterization of germline variants found among Norwegian familial CRC individuals

Patient_ID	VUS (Class 3)	Benign or Likely Benign variants (Class 1 or 2)	Polymorphisms	Total variants/patient
3222	*NOTCH3* NM_000435: c.5854G >A, p.V1952 M(rs115582213)^b^ *POLE* NM_006231: c.3046G > A, p.V1016 M (rs147692158)	*ATM* NM_000051: c.5071A > C, p.S1691R (rs1800059) *BRCA1* NM_007300: c.5019G > A, p.M1673I (rs1799967) *PALB2* NM_024675: c.2993G > A, p.G998E (rs45551636) *PALB2* NM_024675: c.2014G > C, p.E672Q (rs45532440)	rs459552, rs659243, rs2240308, rs1799966, rs16942, rs16941, rs169547, rs4986764, rs1805107, rs506504, rs832582, rs5868032, rs1042821, rs3219484, rs1044009, rs152451, rs2228006, rs1805321, rs4796033, rs1042522, rs861539, rs13125836	28
3308	*NBN* NM_002485: c.1720 T > A, p.L574I (rs142334798)*POLE* NM_006231*:*c.4523G > A, p.R1508H (rs142508245)	*BARD1*N M_000465: c.1075_1095del, p.L359-P365delLPECSSP (rs28997575)	rs459552, rs659243, rs11528010, rs144848, rs169547, rs4986764, rs1805107, rs506504, rs702689, rs832582, rs5868032, rs1799977, rs1042821, rs3219484, rs1805794, rs1044009, rs2228006, rs5744934, rs5744751, rs4796033, rs1042522	24
3387	na	*CDKN2A*N M_000077: c.442G > A, p.A148T (rs3731249)	rs459552, rs659243, rs1801516, rs2240308 rs2070094, rs2229571, rs11528010, rs144848, rs169547, rs4986764, rs1805107, rs506504, rs1126497, rs702689, rs832582, rs5868032, rs3219489, rs1044009, rs2228006, rs1805321, rs5744934, rs1042522	23
3426	na	na	rs459552, rs659243, rs1801516, rs2240308, rs2070094, rs2229571, rs1048108, rs11528010, rs144848, rs169547, rs4986764, rs1805107, rs506504, rs1126497, rs702689, rs832582, rs5868032, rs1805794, rs1044009, rs2228006, rs1805321, rs1042522, rs13125836	23
4932	*NOTCH3* NM_000435: c.5854G >A, p.V1952 M (rs115582213)^a^ *STK11*NM_00045*:*c.841C > A, p.P281T (rs377208033)	na	rs459552, rs659243, rs1801516, rs2240308, rs2229571, rs11528010, rs1799966, rs16942, rs16941, rs799917, rs144848, rs169547, rs1805107, rs506504, rs12642536, rs702689, rs832582, rs5868032, rs3219489, rs1805794, rs1044009, rs2228006, rs1805321, rs861539	26
5324	na	na	rs459552, rs659243, rs2240308, rs2070094, rs2229571, rs144848, rs169547, rs4986764, rs1805107, rs506504, rs1126497, rs12642536, rs832582, rs1799977, rs3219489, rs1044009, rs2228006, rs1805321, rs5744934	19
6174	na	*PMS2* NM_000535: c.1454C > A, p.T485 K (rs1805323)	rs459552, rs659243, rs2240308, rs2070094, rs2229571, rs1048108, rs1799966, rs16942, rs16941, rs799917, rs4986850, rs169547, rs4986764, rs1805107, rs506504, rs1126497, rs12642536, rs702689, rs832582, rs5868032, rs1799977, rs3219484, rs1805794, rs1044009, rs2228006, rs1805321, rs1042522, rs861539	29
6977	na	*BARD1* NM_000465: c.1075_1095del, p.L359-P365delLPECSSP (rs28997575) *BARD1*NM_000465: c.1972C > T, p.R658C (rs3738888) *BRCA2* NM_000059: c.9976A > T, p.K3326X (rs11571833)	rs459552, rs659243, rs1801516, rs2240308, rs1799966, rs16942, rs16941, rs799917, rs144848, rs169547, rs4986764, rs1805107, rs506504, rs1126497, rs12642536, rs702689, rs832582, rs3219489, rs1044009, rs2228006, rs5744934, rs1042522, rs861539, rs28908468	27
9876	*PSMC3IP* NM_016556: c.136G > A, p.V46 M (rs757057684) *RAD51B* NM_133509: c.1063G > A, p.A355T (rs61758785)	*RAD51D* NM_002878: c.698A > G, p.E233G (rs28363284)	rs459552, rs659243, rs2240308, rs2070094, rs2229571, rs169547, rs4986764, rs1805107, rs506504, rs702689, rs832582, rs5868032, rs1799977, rs1042821, rs1805794, rs1044009, rs2228006, rs1726801	21
9998	na	*MSH6* NM_000179: c.2633 T > C, p.V878A (rs2020912)	rs459552, rs659243, rs1801516, rs2240308, rs2070094, rs2229571, rs1048108, rs144848, rs169547, rs4986764, rs1805107, rs506504, rs1126497, rs12642536, rs702689, rs832582, rs5868032, rs1799977, rs1044009, rs2228006, rs1805321, rs5744751, rs1042522	24
10,675	na	*PMS2* NM_000535: c.1531A > G, p.T511A (rs2228007)	rs459552, rs659243, rs1801516, rs2070094, rs2229571, rs11528010, rs1799966, rs16942, rs16941rs799917, rs144848, rs169547, rs4986764, rs1805107, rs506504, rs1126497, rs702689, rs832582,rs5868032, rs1799977, rs1805794, rs1044009, rs2228006, rs10254120, rs1042522	26
12,954	*MUTYH* NM_012222: c.812G > A, p.R271Q (rs149866955) *RAD51C* NM_058216: c.790G > A, p.G264S (rs147241704)	*PMS2* NM_000535: c.1789A > T, p.T597S (rs1805318)	rs459552, rs659243, rs2240308, rs2229571, rs144848, rs169547, rs4986764, rs1805107, rs506504, rs12642536, rs702689, rs832582, rs5868032, rs1044009, rs2228006, rs1805321, rs1726801, rs4796033, rs1042522	22
13,072	*BRCA1* NM_007300*:* c.4315C > T, p.L1439F (rs781260818)	*PMS2* NM_000535: c.1531A > G, p.T511A (rs2228007)	rs459552, rs659243, rs1801516, rs2240308, rs2070094, rs2229571, rs11528010, rs1799966, rs16942, rs16941, rs799917, rs144848, rs169547, rs4986764, rs1805107, rs506504, rs1126497, rs12642536, rs702689, rs832582, rs5868032, rs1799977, rs1805794, rs1044009, rs2228006, rs5744751, rs1042522	29
14,930	na	*PALB2* NM_024675: c.925A > G, p.I309V (rs3809683)	rs459552, rs659243, rs2240308, rs2070094, rs2229571, rs1048108, rs1799966, rs16942, rs16941, rs799917, rs4986850, rs169547, rs4986764, rs1805107, rs506504, rs1126497, rs12642536, rs702689, rs832582, rs5868032, rs1799977, rs3219489, rs1805794, rs1044009, rs152451, rs2228006, rs1805321, rs5744934, rs1042522	30
18,843	*MSH6* NM_000179.2: c.2195G > A, p.R732Q (rs749746725)	*BRCA1* NM_007300: c.3119G > A, p.S1040 N (rs4986852)	rs459552, rs659243, rs2240308, rs2070094, rs2229571, rs11528010, rs144848, rs169547, rs4986764, rs1805107, rs506504, rs1126497, rs12642536, rs832582, rs5868032, rs1799977, rs3219489, rs1044009, rs2228006, rs1805321, rs5744934, rs5744751, rs1042522, rs861539	26
19,411	na	*BARD1* NM_000465: c.1972C > T, p.R658C (rs3738888) *PALB2* NM_024675: c.1010 T > C, p.L337S (rs45494092)	rs459552, rs659243, rs2240308, rs2070094, rs2229571, rs1799966, rs16942, rs16941, rs799917, rs4986850, rs144848, rs169547, rs1805107, rs506504, rs1126497, rs12642536, rs702689, rs832582, rs5868032, rs1805794, 1,044,009, rs2228006, rs1805321, rs5744751, rs1042522, rs861539	28
19,673	*AXIN2* NM_004655: c.344A > G, p.N115S (rs370257532)	*APC* NM_001127510: c.7504G > A, p.G2502S (rs2229995) *PMS2* NM_000535: c.1454C > A, p.T485 K (rs1805323)	rs459552, rs659243, rs2240308, rs2070094, rs2229571, rs1799966, rs16942, rs16941, rs799917, rs144848, rs169547, rs4986764, rs1805107, rs506504, rs702689, rs832582, rs5868032, rs1042821, rs1805794, rs1044009, rs2228006, rs1805321, rs4796033, rs1042522, rs861539, rs3218536	29
20,612	na	*CDKN2A* NM_000077: c.442G > A, p.A148T (rs3731249)	rs459552, rs659243, rs2240308, rs2070094, rs2229571, rs11528010, rs1799966, rs16942, rs16941, rs799917, rs169547, rs4986764, rs1805107, rs506504, rs12642536, rs702689, rs5868032, rs1799977, rs1805794, rs1044009, rs2228006, rs1805321, rs10254120, rs5744934, rs4796033, rs1042522, rs861539	28
21,368	*MAP3K1* NM_005921: c.764A > G, p.N255S (rs56069227)	*ATM* NM_000051: c.2572 T > C, p.F858 L (rs1800056)	rs459552, rs659243, rs2240308, rs2229571, rs11528010, rs1799950, rs169547, rs1805107, rs506504, rs1126497, rs702689, rs832582, rs5868032, rs1799977, rs1044009, rs2228006, rs1805321, rs5744934, rs4796033, rs1042522	22
22,295	na	*BRCA1* NM_007300: c.5019G > A, p.M1673I (rs1799967) *BRIP1* NM_032043: c.890A > G, p.K297R (rs28997570)	rs459552, rs659243, rs2240308, rs2070094, rs2229571, rs1048108, rs1799966, rs16942, rs16941, rs799917, rs144848, rs169547, rs1805107, rs506504, rs12642536, rs702689, rs832582, rs5868032, rs1799977, rs1042821, rs3219489, rs1805794, rs1044009, rs2228006, rs1805321, rs5744934, rs1042522, rs861539	30
23,761	na	na	rs459552, rs659243, rs2240308, rs2070094, rs2229571, rs11528010, rs1799966, rs16942, rs16941, rs799917, rs1799950, rs144848, rs169547, rs4986764, rs1805107, rs506504, rs1126497, rs702689, rs832582, rs5868032, rs1799977, rs1044009, rs2228006, rs1805321, rs1802683, rs1042522	26
23,910	na	*BRCA2* NM_000059: c.6100C > T, p.R2034C (rs1799954) *MSH2* NM_000251: c.965G > A, p.G322D (rs4987188) *MSH6* NM_000179: c.2633 T > C, p.V878A (rs2020912) *PALB2* NM_024675: c.2794G > A, p.V932 M (rs45624036) *PMS2* NM_000535: c.1454C > A, p.T485 K (rs1805323) *BARD1* NM_000465: c.1670G > C, p.C557S (rs28997576)	rs459552, rs659243, rs2240308, rs2070094, rs2229571, rs11528010, rs169547, rs4986764, rs1805107, rs506504, rs1126497, rs702689, rs832582, rs5868032, rs1799977, rs1042821, rs1805794, rs2228006, rs10254120, rs1042522, rs13125836, rs3218536	28
24,140	na	*BRCA1* NM_007300: c.5019G > A, p.M1673I (rs1799967) *PMS2* NM_000535: c.1531A > G, p.T511A (rs2228007)	rs459552, rs659243, rs2240308, rs2070094, rs2229571, rs1048108, rs1799966, rs16942, rs16941, rs799917, rs144848, rs169547, rs1805107, rs506504, rs1126497, rs702689, rs832582, rs5868032, rs1799977, rs1042821, rs3219489, rs1805794, rs1044009, rs2228006, rs10254120, rs5744751, rs1042522	29
24,447	*CHEK2* NM_007194: c.74 T > C, p.V25A (rs587780188)	*NOTCH3* NM_000435: c.3399C > A, p.H1133Q (rs112197217)	rs459552, rs659243, rs1801516, rs2240308, rs2070094, rs2229571, rs1799966, rs16942, rs16941, rs799917, rs169547, rs1805107, rs506504, rs1126497, rs702689, rs832582, rs5868032, rs1805794, rs1044009, rs2228006, rs1805321, rs1042522	24
11,705	*ATM* NM_000051: c.4375G > A, p.G1459R (rs145667735) *MSH2* NM_000251: c.1284C > G, p.H428Q (rs776034412)	*MSH2* NM_000251: c.965G > A, p.G322D (rs4987188) *PMS2* NM_000535: c.1454C > A, p.T485 K (rs1805323)	rs459552, rs659243, rs1801516, rs2229571, rs11528010, rs169547, rs4986764, rs1805107, rs506504, rs1126497, rs702689, rs832582 rs5868032, rs1799977, rs3219489, rs1805794, rs1044009, rs2228006, rs1805321, rs5744751, rs1042522, rs861539, rs13125836	27
12,673	na	*ATM* NM_000051: c.2572 T > C, p.F858 L (rs1800056) *PMS2* NM_000535: c.1454C > A, p.T485 K (rs1805323)	rs459552, rs659243, rs2240308, rs2070094, rs2229571, rs1048108, rs1799950, rs169547, rs4986764, rs1805107, rs506504, rs1126497, rs12642536, rs702689, rs832582, rs5868032, rs1799977, rs1044009, rs2228006, rs1805321, rs1802683, rs4796033, rs1042522, rs861539	26
13,393	*NBN* NM_002485.4: c.643C > T, p.R215W (rs34767364)	*BARD1* NM_000465: c.1972C > T, p.R658C (rs3738888) *BRIP1* NM_032043: c.577G > A, p.V193I (rs4988346) *PMS2* NM_000535: c.1531A > G, p.T511A (rs2228007) *ATM* NM_000051: c.4258C > T, p.L1420F (rs1800058) *NOTCH3* NM_000435.2: c.3058G > C, p.A1020P (rs35769976) *NOTCH3* NM_000435: c.3547G > A, p.V1183 M (rs10408676)	rs459552, rs659243, rs2240308, rs2070094, rs2229571, rs1799966, rs16942, rs16941, rs799917, rs169547, rs4986764, rs1805107, rs506504, rs702689, rs832582, rs5868032, rs1805794, rs1044009, rs2228006, rs1805321, rs1042522	28
14,963	*PALB2* NM_024675: c.232G > A, p.V78I (rs515726085)	*PALB2* NM_024675: c.2590C > T, p.P864S (rs45568339) *STK11* NM_000455: c.1062C > G, p.F354 L (rs59912467)	rs659243, rs1801516, rs2240308, rs2070094, rs2229571, rs11528010, rs1799966, rs16942, rs16941, rs799917, rs1799950, rs144848, rs169547, rs4986764, rs1805107, rs506504, rs1126497, rs12642536, rs702689, rs5868032, rs1799977, rs3219489, rs1805794, rs1044009, rs2228006, rs1805321, rs5744751, rs1042522, rs861539, rs3218536	33
19,609^a^	na	*BRCA2* NM_000059: c.4258G > T, p.D1420Y (rs28897727) *POLE* NM_006231: c.2083 T > A, p.F695I (rs5744799)	rs459552, rs659243, rs2240308, rs2229571, rs1799966, rs16942, rs16941, rs799917, rs144848, rs169547, rs4986764, rs1805107, rs506504, rs702689, rs832582, rs5868032, rs1799977, rs3219484, rs1805794, rs1044009, rs2228006, rs1805321, rs5744934, rs1042522, rs13125836	28
22,953	*NOTCH3* NM_000435: c.5208G > C, p.E1736D (rs200331646) *MSH2* NM_000251: c.128A > G, p.Y43C (rs17217723) *RAD51B* NM_133510: c.515 T > G, p.L172 W (rs34094401)	*BRCA2* NM_000059: c.2971A > G, p.N991D (rs1799944) *CDH1* NM_004360: c.1774G > A, p.A592T (rs35187787)	rs459552, rs659243, rs2240308, rs2229571, rs11528010, rs1799966, rs16942, rs16941, rs799917, rs169547, rs4986764, rs1805107, rs506504, rs12642536, rs702689, rs832582, rs5868032, rs1042821, rs3219489, rs1805794, rs1044009, rs2228006, rs1805321, rs1726801	29
24,789	*APC* NM_001127510*:* c.4334C > T, p.T1445I (rs760686348) *PALB2* NM_024675: c.1250C > A, p.S417Y (rs45510998)	*BARD1* NM_000465: c.1972C > T, p.R658C (rs3738888) *PALB2* NM_024675: c.2993G > A, p.G998E (rs45551636) *PALB2* NM_024675: c.2014G > C, p.E672Q (rs45532440) *POLE* NM_006231: c.776G > A, p.R259H (rs61732929) *NOTCH3* NM_000435: c.3399C > A, p.H1133Q (rs112197217)	rs459552, rs659243, rs2240308, rs2229571, rs1799966, rs16942, rs16941, rs799917, rs169547, rs4986764, rs1805107, rs506504, rs1126497, rs12642536, rs702689, rs832582, rs5868032, rs1799977, rs3219489, rs1044009, rs152451, rs2228006, rs1042522	30
25,167	*NBN* NM_002485.4: c.643C > T, p.R215W (rs34767364)	*ATM* NM_000051: c.2119 T > C, p.S707P (rs4986761)	rs459552, rs659243, rs2240308, rs2229571, rs11528010, rs169547, rs4986764, rs1805107, rs506504, rs12642536, rs702689, rs832582, rs5868032, rs1799977, rs1805794, rs2228006, rs1805321, rs1726801, rs4796033, rs1042522	22
5597	*MAP3K1* NM_005921: c.2816C > G, p.S939C (rs45556841)	*ATM* NM_000051: c.5071A >C, p.S1691R (rs1800059)	rs459552, rs659243, rs1801516, rs2070094, rs2229571, rs11528010, rs1799966, rs16942, rs16941, rs799917, rs4986850, rs144848, rs169547, rs4986764, rs1805107, rs506504, rs1126497, rs12642536, rs702689, rs832582, rs5868032, rs1042821, rs1805794, rs1044009, rs2228006, rs1805321, rs1042522	29
8913	*RAD51B* NM_133509: c.1063G > A, p.A355T (rs61758785) *EPCAM* NM_002354: c.267G > C, p.Q89H (rs146480420)	*PMS2* NM_000535: c.1454C > A, p.T485 K (rs1805323)	rs459552, rs659243, rs2240308, rs2070094, rs2229571, rs1048108, rs11528010, rs1799966, rs16942, rs16941, rs799917, rs144848, rs169547, rs4986764, rs1805107, rs506504, rs1126497, rs702689, rs832582, rs5868032, rs1805794, rs1044009, rs2228006, rs5744751, rs1042522, rs861539, rs13125836	30

^a^ Recently classified as Benign by ACMG Guidelines, 2015

^b^ Patient ID carrying *CHEK2* c.470 T > C, p.I157T

Importantly, we identified a likely pathogenic variant in a moderate-penetrance gene (*CHEK2* c.470 T > C, p.I157T) in a female patient diagnosed with colon cancer at 42 years, melanoma at 44 years and BC at 57 years with a proficient IHC MMR profile and fulfilling the revised Bethesda guidelines (Patient 19,609) (Table 1).

The *CHEK2* c.470 T > C has been classified as pathogenic according to the ACMG guidelines [51], and has a lower allele frequency (1.89*10–3) in the Norwegian population, compared to the non-Finnish European population (5.4*10–3) (http://norgene.no/vcf-miner/ and gnomAD database, respectively) [34, 35, 50]. The variant is reported in ClinVar as “conflicting interpretations of pathogenicity, risk factor” (Variation ID: 5591). When the revised Bethesda guidelines were considered, the mutation detection rate was thus 4.8% (1/21).

Overall, 25 unique VUS were found in 18 out of the 34 patients (Table 2). The detected VUS were distributed among 17 different genes: *MAP3K1* (in 2 patients)*, NBN* (in 3 patients), *NOTCH3* (in 3 patients), *RAD51B* (in 3 patients), *MSH2* (in 2 patients), *PALB2* (in 2 patients), *POLE* (in 2 patients) and the remaining were found in *APC, ATM*, *AXIN2*, *BRCA1, CHEK2*, *EPCAM*, *MSH6, MUTYH*, *RAD51C* and *STK11* (Table 2). The minor allele frequency (MAF) values of these variants were very low or no frequency data have been reported.

### Protein and splicing-dedicated in silico analyses

The 25 unique VUS were analyzed by using five in silico prediction tools with different underlying algorithms to estimate the impact of the variants on the structure and function of the corresponding proteins.

Concordances between the 5 prediction tools were found for 2 out of the 25 VUS, suggesting a potentially damaging effect on protein level for the variants: *MUTYH* c.812G > A (p.R271Q) and *MSH2* c.128A > G (p.Y43C) (Table 3). In the other hand, 6 out of 25 VUS were consistently predicted as benign: *NBN* c.1720 T > A (p.L574I),

**Table 3:** In silico data obtained for the variants of unknown significance (VUS) identified in our study of familial CRC individuals

Selected variants (VAR)						Reference splice site-dedicated analyses								Cryptic splice site-dedicated analyses			ESR-dedicated analyses			**Protein-dedicated analyses**				
Patient_ID	Genomic position (GRCh37)	Gene	Exon	Nucleotide change (cNomen)	Predicted protein change (pNomen)	Nearest reference		MES scores			SSFL scores			Potential local splice effect	Local MES scores		∆tESRseq	**∆Hzei**	**ΔΨ**					
						splice site		WT	Var	VAR vs WT	WT	Var	VAR vs WT		WT	Var								
						Distance	Type						**∆ (%)**											
						(nt)	(3’ or 5’ss)			**Δ (%)**										**AGVGD**	**SIFT**	**MAPP**	**PolyPhen-2**	**MutationTaster**
3222 & 4932	chr19:15273335 C>T	***NOTCH3***	**32**	**c.5854G>A**	**p.V1952M**	39	3’	11.5	11.5	0	89.2	89.2	0	-	-	-	**-1.78501**	**-11.15**	**-0.89**	C15	Deleterious	bad	probably damaging	disease causing
3222		*POLE*	25	c.3046G>A	p.V1016M	-15	5’	9.1	9.1	0	82.5	82.5	0	-	-	-	-0.74478	1	-0.43	C0	Deleterious	bad	benign	disease causing
3308	chr8:90965597 A>T	*NBN*	11	c.1720T>A	p.L574I	-126	5’	8.0	8.0	0	82.4	82.4	0	-	-	-	1.97427	33.38	0.18	C0	Tolerated	good	benign	polymorphism
	chr12:133219838 C>T	*POLE*	35	c.4523G>A	p.R1508H	-29	5’	7.9	7.9	0	73.8	73.8	0	-	-	-	-0.600279	-3.17	-0.08	C0	Tolerated	good	benign	disease causing
4932	chr19:1221318 C>A	*STK11*	6	c.841C>A	p.P281T	-22	5’	6.0	6.0	0	79.9	79.9	0	-	-	-	-0.17437	7.7	-0.72	C0	Tolerated	good	benign	disease causing
5597	chr5:56177843 C>G	*MAP3K1*	14	c.2816C>G	p.S939C	447	3’	12.0	12.0	0	100.0	100.0	0	-	-	-	-0.486881	-16.1	0	C0	Deleterious	good	benign	polymorphism
9876	chr17: 40729320 C>T	*PSMC3IP*	3	c.136G>A	p.V46M	1	3’	12.5	11.7	-6	87.5	83.6	-4	-	-	-	2.0413	74.24	-0.3	C0	Deleterious	-	possibly damaging	disease causing
9876 & 8913	chr14:69061228 G>A	*RAD51B*	11*	c.1063G>A	p.A355T	27	3’	11.8	11.8	0	80.2	80.2	0	-	-	-	-1.24035	-50.64	-	C0	Deleterious	-	benign	polymorphism
12954	chr1: 45797950_C>T	*MUTYH*	10	c.812G>A	p.R271Q	33	3’	9.5	9.5	0	86.8	86.8	0	-	-	-	-2.31042	0.88	0.09	C35	Deleterious	bad	possibly damaging	disease causing
	chr17:56787304 G>A	*RAD51C*	5	c.790G>A	p.G264S	-48	5’	8.6	8.6	0	75.4	75.4	0	New Acceptor Site?	0	2.5	-1.71397	-59.31	-0.21	C0	Tolerated	good	benign	disease causing
13072	chr17:41234463 G>A	*BRCA1*	12	c.4315C>T	p.L1439F	-43	5’	6.6	6.6	0	85.2	85.2	0	-	-	-	-0.261141	8.83	-2.67	C0	Tolerated	good	benign	polymorphism
18843	chr2:48027317 G>A	*MSH6*	4**	c.2195G>A	p.R732Q	-978	5’	8.9	8.9	0	81.6	81.6	0	-	-	-	-0.805417	-13.15	0	C0	Tolerated	good	benign	disease causing
19673	chr17:63554395 T>C	*AXIN2*	2	c.344A>G	p.N115S	460	3’	11.1	11.1	0	93.3	93.3	0	New Acceptor Site?	11.1	7.2	-0.162849	-2.19	-0.05	C0	Tolerated	good	benign	disease causing
21368	chr5:56155672 A>G	***MAP3K1***	**3**	**c.764A>G**	**p.N255S**	-71	5’	7.5	7.5	0	78.5	78.5	0	New Acceptor Site?	**4.7**	**8.8**	-1.18661	6.7	-0.04	C0	Tolerated	good	benign	polymorphism
24447	chr22:29130636 A>G	*CHEK2*	2	c.74T>C	p.V25A	80	3’	1.7	1.7	0	85.5	85.5	0	-	-		-0.166788	36.67	-0.07	C0	Tolerated	good	benign	polymorphism
11705	chr11:108160467 G>A	*ATM*	29	c.4375G>A	p.G1459R	-62	5’	8.9	8.9	0	87.5	87.5	0	-	-		0.552026	-27.26	-0.09	C15	Deleterious	bad	probably damaging	disease causing
	chr2:47672694 C>G	*MSH2*	8	c.1284C>G	p.H428Q	8	3’	10.1	10.1	0	87.3	87.3	0	-	-		0.703136	22.05	0.05	C0	Tolerated	good	possibly damaging	disease causing
13393 & 25167	chr:8_90983460 G>A	*NBN*	6	c.643C>T	p.R215W	59	3’	6.2	6.2	0	86.8	86.8	0	-	-		-1.00071	-16.87	-0.09	C0	Tolerated	good	probably damaging	polymorphism
14963	chr16:23647635 C>T	*PALB2*	4**	c.232G>A	p.V78I	21	3’	10.0	10.0	0	90.3	90.3	0	-	-		-0.783039	-29.17	0.8	C0	Tolerated	good	benign	polymorphism
22953	chr19:15278214 C>G	*NOTCH3*	29	c.5208G>C	p.E1736D	9	3’	7.8	7.8	0	84.2	84.2	0	-	-		0.0501789	-10.44	0.22	C35	Deleterious	bad	benign	disease causing
	chr2:47630458 A>G	*MSH2*	1*	c.128A>G	p.Y43C	-84	5’	10.1	10.1	0	90.6	90.6	0	New Donor Site?	0	1.2	1.50425	4.7	2.11	**C55**	Deleterious	bad	probably damaging	disease causing
	chr14:68352648 T>G	*RAD51B*	6	c.515T>G	p.L172W	-58	5’	9.5	9.5	0	83.7	83.7	0	-	-		1.62772	67.77	1.31	C0	Deleterious	bad	possibly damaging	polymorphism
24789	chr5:112175625 C>T	*APC*	15*	c.4334C>T	p.T1445I	2376	3’	7.5	7.5	0	93.6	93.6	0	-	-		-1.61023	-90.13	-	C0	Tolerated	good	benign	polymorphism
	chr16:23646617 G>T	*PALB2*	4**	c.1250C>A	p.S417Y	-435	5’	8.9	8.9	0	87.5	87.5	0	-	-		-0.551724	-22.83	0.05	C0	Deleterious	bad	probably damaging	disease causing
8913	chr2:47601029 G>C	*EPCAM*	3	c.267G>C	p.Q89H	83	3’	7.0	7.0	0	90.1	90.1	0	-	-		-0.40237	-28.93	0	C0	Tolerated	bad	possibly damaging	disease causing

In order to predict the biological impact of the 25 VUS, RNA splicing- and protein-dedicated bioinformatics analyses were performed as described under Materials and Methods. The stars indicate exons that could not be tested in our minigene assay, either because of their terminal position (*, 1st or last exons) or because of their large size (**). Results shown in bold were considered as predictive of a potential variant-induced negative biological effect. MES, MaxEntScan; SSFL, Splice Site Finder-Like; nt, nucleotide; 3’ or 5’ss, 3’ splice site or 5’ splice site; ESR, exonic splicing regulators; AGVGD, align-GVGD (C0, C15, C25, C35, C.45, C55, or C65 with C65 most likely to interfere with function and C0 least likely), SIFT, Sorting Intolerant From Tolerant (tolerated or deleterious), MAPP, Multivariate Analysis of Protein Polymorphism (good or bad), PolyPhen-2, Polymorphism Phenotyping v2 (benign, possibly damaging or probably damaging), MutationTaster (polymorphism or disease causing), CRC: colorectal cancer.

In order to predict the biological impact of the 25 VUS, RNA splicing- and protein-dedicated bioinformatics analyses were performed as described under Materials and Methods. The stars indicate exons that could not be tested in our minigene assay, either because of their terminal position (*, 1st or last exons) or because of their large size (**). Results shown in bold were considered as predictive of a potential variant-induced negative biological effect. MES, MaxEntScan; SSFL, Splice Site Finder-Like; nt, nucleotide; 3′ or 5’ss, 3′ splice site or 5′ splice site; ESR, exonic splicing regulators; AGVGD, align-GVGD (C0, C15, C25, C35, C.45, C55, or C65 with C65 most likely to interfere with function and C0 least likely), SIFT, Sorting Intolerant From Tolerant (tolerated or deleterious), MAPP, Multivariate Analysis of Protein Polymorphism (good or bad), PolyPhen-2, Polymorphism Phenotyping v2 (benign, possibly damaging or probably damaging), MutationTaster (polymorphism or disease causing), CRC: colorectal cancer

In order to predict their biological impact, RNA splicing- and protein-dedicated bioinformatics analyses were performed as described under Materials and Methods. Results shown in bold were considered as predictive of a potential variant-induced negative biological effect. MES, MaxEntScan; SSFL, Splice Site Finder-Like; nt, nucleotide; 3′ or 5’ss, 3′ splice site or 5′ splice site; ESR, exonic splicing regulators; AGVGD, align-GVGD (C0, C15, C25, C35, C.45, C55, or C65 with C65 most likely to interfere with function and C0 least likely), SIFT, Sorting Intolerant From Tolerant (tolerated or deleterious), MAPP, Multivariate Analysis of Protein Polymorphism (good or bad), PolyPhen-2, Polymorphism Phenotyping v2 (benign, possibly damaging or probably damaging), MutationTaster (polymorphism or disease causing)

*BRCA1* c.4315C > T (p.L1439F), *MAP3K1* c.764A > G (p.N255S), *CHEK2* c.74 T > C (p.V25A), *PALB2* c.232G > A (p.V78I) and *APC* c.4334C > T (p.T1445I). Discrepancies were pronounced for the variants in the *POLE* (*n* = 2)*, STK11, MAP3K1, PSMC3IP, RAD51C, MSH6, AXIN2, MSH2, NBN, NOTCH3, RAD51B, PALB2* and *EPCAM* genes (Table 3).

Two out of the 25 VUS were bioinformatically predicted to affect RNA maturation by potentially modifying splicing signals (Table 3). More specifically, according to our in silico results, *NOTCH3* c.5854G >A (identified in Patients 3222 and 4932) was predicted to potentially induce exon 32 skipping by alteration of exonic splicing regulatory elements, whereas *MAP3K1* c.764A > G (detected in Patient 21,368) was predicted to introduce a deletion of the first 131 nucleotides of exon 3 (r.634_764del) due to the creation of a putative new acceptor splice site. Skipping of *NOTCH3* exon 32 would produce a transcript with a frameshift deletion of 98 nucleotides (*NOTCH3* r.5816_5913del), potentially leading to the production of a carboxy-terminally truncated NOTCH3 protein p.(Lys1940Glyfs*14). The *MAP3K1* r.634_764del transcript would be expected to be degraded by nonsense mediated decay and/or result in a very short MAP3K1 protein p.(Val212Leufs*45). The *NOTCH3* c.5854G >A was identified in two patients (Patients 3222 and 4932) that fulfilled the revised Bethesda guidelines and AMS criteria, respectively while the *MAP3K1* c.764A > G (Patient 21,368) in a patient which family fulfilled the revised Bethesda guidelines (Table 1).

### Minigene splicing assays

Because patient RNA was not available, we decided to experimentally assess the impact of these 2 variants (*NOTCH3* c.5854G >A and *MAP3K1* c.764A > G) might have on RNA splicing by performing cell-based minigene splicing assays.

**Fig. 1:**
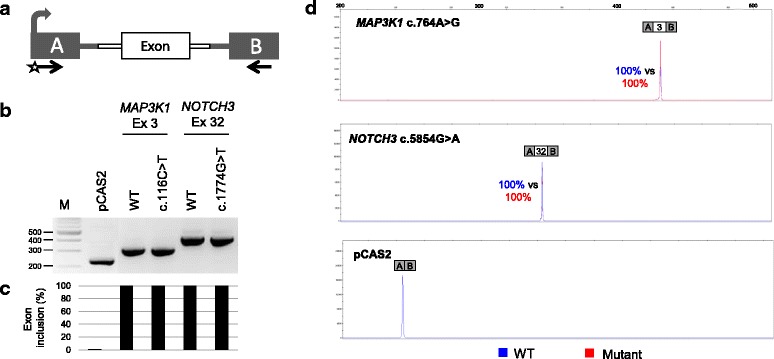
Evaluation of variant-induced splicing alterations by using a cell-based minigene assay. **a** Structure of pCAS2 minigenes used in the splicing reporter assay. The bent arrow indicates the CMV promoter, boxes represent exons, lines in between the boxes indicate introns, and arrows below the exons represent primers used in RT-PCR reactions. The minigenes were generated by inserting a genomic fragment containing the exon of interest together with its flanking intronic sequences into the intron of pCAS2, as described under Materials and Methods. **b** Analysis of the splicing pattern of pCAS2 minigenes carrying variants identified in this study. Wild-type (WT) and mutant constructs, as indicated, were introduced into HeLa cells and the transcripts of the minigenes were analyzed by RT-PCR 24 h post-transfection. The image shows the results of a representative experiment in which the RT-PCR products were separated on a 2.5% agarose gel stained with EtBr and visualized by exposure to ultraviolet light. M, 100 bp DNA ladder (New England Biolabs). **c** Quantification of splicing events observed in the minigene splicing assay. The relative levels of exon inclusion indicated under the gel are based on RT-PCR experiments equivalent to those shown in B but performed with a fluorescent forward primer and then separated on an automated sequencer. Quantification results were obtained by using the GeneMapper v5.0 software (Applied Biosystems) and correspond to the average of two independent fluorescent-RT-PCR experiments. **d** Representative fluorescent RT-PCR experiment. The panel shows superposed peaks corresponding to the WT and mutant products (in blue and red, respectively), as indicated

As shown in Fig. 1 we found that *NOTCH3* c.5854G >A and *MAP3K1* c.764A > G did not modify the splicing pattern of the minigenes’ transcripts. These data thus disagree with the in silico predictions and suggest that either the exon 32 of *NOTCH3* and the exon 3 of *MAP3K1* are refractory to splicing mutations (the predictions thus being incorrect) or that the minigenes used in our study do not fully reproduce the splicing pattern of the mutant exons in *NOTCH3* and *MAP3K1* bona fide transcripts (the predictions being eventually correct). Complementary studies using RNA from *NOTCH3* c.5854G >A and *MAP3K1* c.764A > G carriers need to be performed to verify the pertinence of these results.

## Discussion

The major unexpected finding in our Norwegian high-risk CRC cohort was the detection of a likely pathogenic variant in *CHEK2* (c.470 T > C, p.I157T)*,* a moderate-penetrance gene not traditionally associated with CRC, in an individual with a LS-evocative personal/family history and a high number of Class 3 variants in BC- and CRC- associated genes. Interestingly, the *CHEK2* (c.470 T > C, p.I157T) has an allele frequency of 1.89*10–3 in the Norwegian population (http://norgene.no/vcf-miner/), and is reported in ClinVar as having conflicting interpretations of pathogenicity/being a risk factor (Variation ID: 5591). Importantly, there is no systematic classification for most of the genetic variants found by NGS, and, in more general terms, the impact of low- to moderate-penetrance pathogenic variants with respect to clinical management is not fully understood [52]. Co-segregation or case-control studies for further evaluation will be key in understanding whether such germline variant may have a modifying effect, since we do not yet have evidence-based guidelines for the majority of these genes.

On the other hand, *CHEK2* germline variants have been described to confer an elevated risk of BC (relative risk = 3.0) [53]. However, the presence of pathogenic variants in *CHEK2* is not frequently associated with cancer in high-risk BC families, prompting speculation that there may be several low-penetrance or moderate-penetrance BC risk genes segregating independently within these families [23, 54, 55]. Co-segregation analyses may add clues in our understanding whether this germline variant is implicated in CRC predisposition. Finally, we did not find pathogenic variants in *POLE* in our cohort, which is in contrast to what has been described in families with high burden of CRC adenomas and carcinomas in addition to extra-colonic cancers [56].

According to the Prospective LS Database (PLSDB), a total of 125 Norwegian families had a demonstrated pathogenic variant in either *MLH1* (*n* = 21), *MSH2* (*n* = 52), *MSH6* (*n* = 36), or *PMS2* (*n* = 16) [25]. On the other hand, a large portion of high-risk CRC families without pathogenic variant in MMR or *EPCAM* genes may be explained by a polygenic model involving a combination of multiple genomic risk factors, including the effect of either low-penetrance susceptibility alleles [57], high-penetrance genes which have not been tested, or the effect of environmental factors. In addition, emerging data suggest that CRC cases negative for pathogenic MMR variants may contain a significantly higher number of copy-neutral loss of heterozygosity (cnLOH) regions, some located within well-known oncogenes and tumor suppressor genes, compared to cases of sporadic CRC [58]. These genomic variations, which were not investigated in this study, may provide an additional explanation for high-risk CRC phenotypes without MMR or *EPCAM* pathogenic variants.

Recent NGS studies described the presence of heterozygous pathogenic *BRCA1/2* or *APC* variants as well as biallelic *MUTYH* alterations in individuals with clinical features resembling those of LS [5, 22]. More precisely, those studies reported that 7% of patients with CRC carried pathogenic variants in non-LS genes, including 1.0% with *BRCA1/2* mutations, and nearly two thirds of probands with high-penetrance non-LS mutations lacked clinical histories suggestive of their respective syndromes [5].

From 34 high-risk CRC individuals, our NGS panel testing identified one patient that carried a pathogenic variant in a gene with reportedly moderate penetrance. Our finding is in line with the mutation frequency (6%) in non-LS cancer susceptibility genes for individuals undergoing LS genetic testing [21] and 4% of patients with BC tested negative for *BRCA1/2* genes [23]. Our results may have implications for an appropriate genetic counseling and follow-up of the patients and family members.

Besides the likely pathogenic *CHEK2* variant, we identified a total of 25 variants in our cohort for which there were not so much data as to their clinical significance. We thus undertook bioinformatics analyses in an attempt to predict the biological impact of these Class 3 variants, both at the RNA and protein level, the ultimate goals being: (i) to discriminate pathogenic from non-pathogenic alterations in this set of variants and (ii) to further pinpoint the genetic determinants of high risk CRC in our cohort. On one hand, our RNA splicing-dedicated bioinformatics evaluation predicted that 2 out of the 25 VUS identified in this study (*NOTCH3* c.5854G >A, p.V1952 M and *MAP3K1* c.764A > G, p.N255S) could potentially affect RNA splicing. These two variants were then experimentally analyzed by performing minigene splicing assay. Our results revealed that neither variant altered the splicing pattern of the representative minigenes, suggesting that they do not affect the splicing of *NOTCH3* or *MAP3K1* transcripts. Additional experiments based on the analysis of RNA from carriers of these variants will be important to verify our minigene results. On the other hand, our protein-dedicated bioinformatics analysis yielded 8 consistent predictions (2 VUS predicted as deleterious and 6 as benign) and several conflicting results that were not explored further.

In this scenario, not only functional tests, but also co-segregation studies will be key to understanding whether the VUS detected in this work are non-pathogenic or otherwise have a causal or a modifying effect. Importantly, we do not yet have evidence-based guidelines for the majority of the genes carrying the VUS identified in this study and, in more general terms, the impact of low- to moderate-penetrance pathogenic variants with respect to clinical management is not fully understood. Most of these variants may in the future be reclassified as deleterious or benign, but in the meantime, they cannot be used to make clinical decisions [59]. Informed (re)classification of VUS in cancer-associated genes may cater to more appropriate risk-management, and may provide significant clues for the identification of additional patients carrying such uncommon variants.

NGS panel testing may benefit patients with a personal or family history compatible with more than one recognized CRC inherited syndrome. The CRC risk management strategy for these individuals is not yet available and there is a need to identify new high-, moderate-, and low- penetrance gene variants that may affect the risk of CRC or LS-associated tumors in non-MMR pathogenic carriers. The identification of such gene variants in combination with family history may contribute to more intensive surveillance and improved prevention [23].

## Conclusions

Our study provides information on genetic locus that might possibly be related to cancer susceptibility, demonstrating that genes presently not routinely tested may be important for capturing cancer predisposition in these patients. In addition, we stratified 25 VUS by the use of RNA splicing- and protein-dedicated in silico analyses. Further studies are necessary for making reliable estimates of cancer risk for the VUS found in this study and allowing appropriate genetic counseling for the patients and their relatives.

Surveillance for early cancer detection is essential to ensure optimal survival for patients afflicted with familial cancers. Our findings pinpoint the need of more studies to unravel the mechanisms underlying the development of CRC in high-risk patients and the identifying for new cancer predisposition genes.

## Additional file

Additional file 1: **Table S1.** Primers used in the pCAS2 minigene splicing assay. (DOCX 15 kb)

## Abbreviations

ACMG: American college of medical genetics and genomics
AMS: Amsterdam criteria
BC: Breast cancer
CRC: Colorectal cancer
IHC: Immunohistochemistry
LS: Lynch syndrome
MAF: Minor allele frequency
MMR: Mismatch repair genes
MSI: Microsatellite instability
NGS: Next generation sequencing
PLSDB: Prospective lynch syndrome database
SNV: Single nucleotide variant
VUS: Variants of unclassified significance
