# Supplementary material for: Identification of genetic variants for clinical management of familial colorectal tumors
Source: BMC Med Genet. 2018 Feb 20;19:26. doi: 10.1186/s12881-018-0533-9 (PMC5819082; doi:10.1186/s12881-018-0533-9)
Supplement: Supplementary file 1 — Table S1. Primers used in the pCAS2 minigene splicing assay. (DOCX 15 kb) [file 12881_2018_533_MOESM1_ESM.docx]

| Purpose | Forward (F) or reverse (R) primers | |
| --- | --- | --- |
|  | Name | Sequence (5’-3’) |
| PCR  (cloning, minigene preparation) | MAP3K1 Ex3 InFus BamHI-F | AAGAAGTGCAGGATCCCAAAATTGTAAGGGAGGAGGTTGC |
|  | MAP3K1 Ex3 InFus MluI-R | TCAAAACAAGACGCGTCAGTAGCTTTACCCACCACTGC |
|  | NOTCH3.Ex32.InFus.BglII-F | AAGAAGTGCAGGATCTGCACAGAGTCATTTTGCAGATTTG |
|  | NOTCH3.Ex32.InFus.Mlu-R | TCAAAACAAGACGCGTACACCCAGCCATTAGGCAC |
| Sequencing of minigene inserts | pCAS-Seq-F | GGGTCAATAGCAGTGAGAGG |
|  | pCAS-Seq-R | GCTCCATTTCACAGGTAGAGA |
| RT-PCR and/or sequencing of RT-PCR products | pCAS-KO1-F | TGACGTCGCCGCCCATCAC |
|  | 6FAM-pCAS-KO1-F (5’-fluo) | TGACGTCGCCGCCCATCAC |
|  | pCAS-2R | ATTGGTTGTTGAGTTGGTTGTC |

Additional file 1: Table S1. Primers used in the pCAS2 minigene splicing assay. BamHI and MluI restriction sites are underlined.
